# Paraneoplastic pemphigus associated with small lymphocytic lymphoma: A case report

**DOI:** 10.1097/MD.0000000000024039

**Published:** 2021-01-15

**Authors:** Dafen Wang, Zhilu Chen

**Affiliations:** The second Clinical Medical College of Zhejiang Chinese Medical University, Hangzhou, China.

**Keywords:** paraneoplastic pemphigus, small lymphocyte lymphoma

## Abstract

**Introduction:**

Paraneoplastic pemphigus (PNP) is a life-threatening autoimmune blistering disease associated with underlying neoplasms. Currently, this disease is very difficult to treat.

**Patient concerns:**

We reported a rare case of paraneoplastic pemphigus associated with small lymphocytic lymphoma responsive to desmoglein 3 (Dsg3) and bullous pemphigoid (BP) antigen 180.

**Diagnoses:**

The initial diagnosis was hypothesized to be Stevens-Johnson syndrome based on the severe mucosal erosion and polymorphous skin lesions. However, the histopathological examination of the skin biopsy and immunology revealed PNP.

**Interventions:**

Anti-tumor therapy, immunosuppression and anti-infective therapy were administered.

**Outcomes:**

After a series of treatments, the skin lesions had been alleviated remarkably. Enzyme-linked immunoassays indices for Dsg3 and bullous pemphigoid antigen 180 decreased (Dsg3, 32; bullous pemphigoid antigen 180, 70.44). Unfortunately, 2 months later, the patient suffered respiratory failure due to the lung impairment of small lymphocytic lymphoma and infection. Eventually, the patient chose to be discharged from the hospital and lost the opportunity for follow-up treatment as he could not afford the expensive treatment costs.

**Lessons:**

It is highly susceptible to misdiagnosis due to polymorphous skin lesions. In this case, it was also initially misdiagnosed as Stevens-Johnson syndrome. Therefore, we should pay great attention to differential diagnosis. When refractory stomatitis and mucosal erosions occur, the possibility of PNP should be considered first. At the same time, pathology, immunology and other related tests as well as the examination of primary tumors should be carried out as soon as possible.

## 1 Introduction

Paraneoplastic pemphigus (PNP) is a rare autoimmune bullous disease associated with benign and malignant tumors such as non-Hodgkin lymphoma, Castleman disease and thymoma.^[1–3]^ PNP is characterized by the presence of autoantibodies against the plakin family proteins (envoplakin, periplakin), desmogleins, desmocollins and bullous pemphigoid antigens.^[4–6]^ The clinical picture of PNP can be extremely diverse and similar to several diseases such as pemphigus, pemphigoid, erythema multiform, graft-versus-host disease, and lichen planus.^[3]^ Therefore, the diagnosis of PNP can be confounding and highly susceptible to misdiagnosis. This is extremely worthy of our close attention. The following, we reported a rare case of PNP which was initially misdiagnosed as Stevens-Johnson syndrome.

## 2 Case presentation

**Figure 1:**
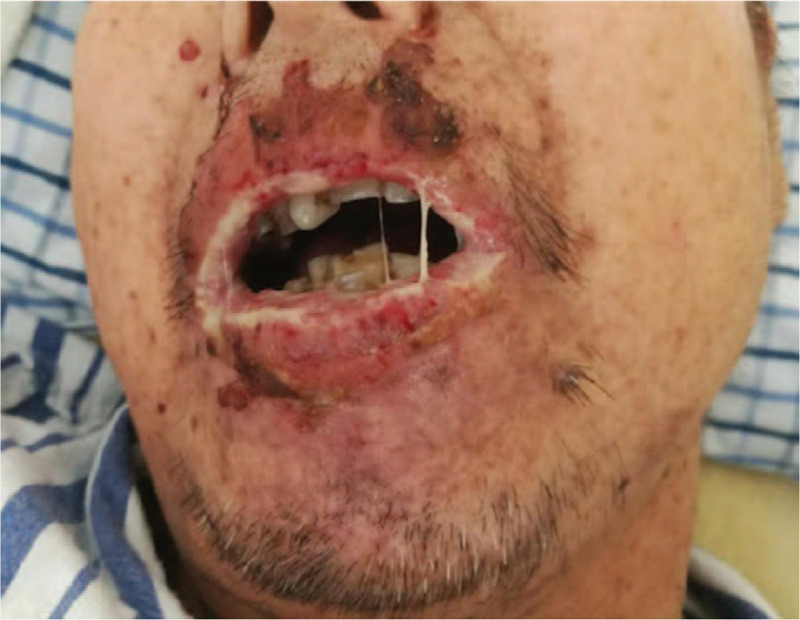
Multiple ulcers in the oral cavity, and lip erosion covered with white secretions.

A man born in 1951 was diagnosed with small lymphocytic lymphoma (SLL) in 2015. He was treated with 6 cycles of R-CHOP chemotherapy (rituximab + cyclophosphamide + epirubicin + vindesine + prednisone). Myelograms every 6 months showed sustained complete remission. In December 2018, SLL recurred and cytofluorimetric analysis showed 8% of the cells were abnormal monoclonal small B-cells expressing CD5(dim), CD20, CD23, CD38, PAX-5, BCL-2, Ki-67(30%). And he received a course of R-GDP chemotherapy (rituximab + gemcitabine + cisplatin + dexamethasone). In early April 2019, erythema, blisters, and erosions appeared on the skin, followed by oral ulcers and lip erosions (Fig. 1). The severe mucosal erosions and polymorphic cutaneous lesions were initially diagnosed as Stevens-Johnson syndrome. Methylprednisolone 40 mg daily and ibrutinib 420 mg daily were administered for 1 week. However, his condition did not improve and the area of skin lesions expanded.

**Figure 2:**
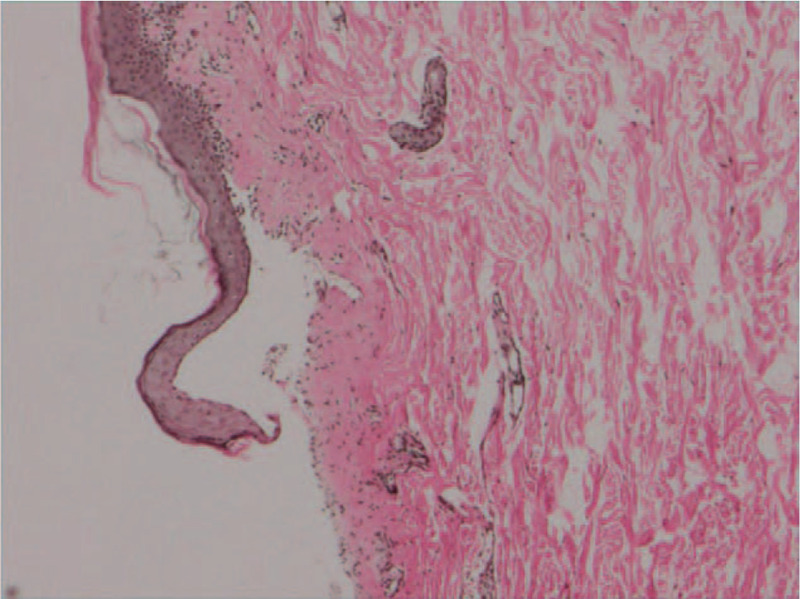
The biopsy of the lesion showed epidermolysis, bullous lesion formation, a few pigmented areas and numerous lymphocytes and plasma cell infiltration around the blisters and the superficial dermis (hematoxylin and eosin, original magnification ×400).

Therefore, the biopsy of the lesion showed epidermolysis, bullous lesion formation, a few pigmented areas and numerous lymphocytes and plasma cell infiltration around the blisters and the superficial dermis (Fig. 2). Immunofluorescence identified epidermis intercellular IgG deposits. Enzyme-linked immunoassays (ELISA) were positive for both desmoglein 3 (Dsg3) (index, 200; cut-off, >20) and bullous pemphigoid antigen 180 (BP180) (index, 188.49; cut-off, >20). The diagnosis of PNP was made on the basis of clinical, histopathological and immunological findings.

**Figure 3:**
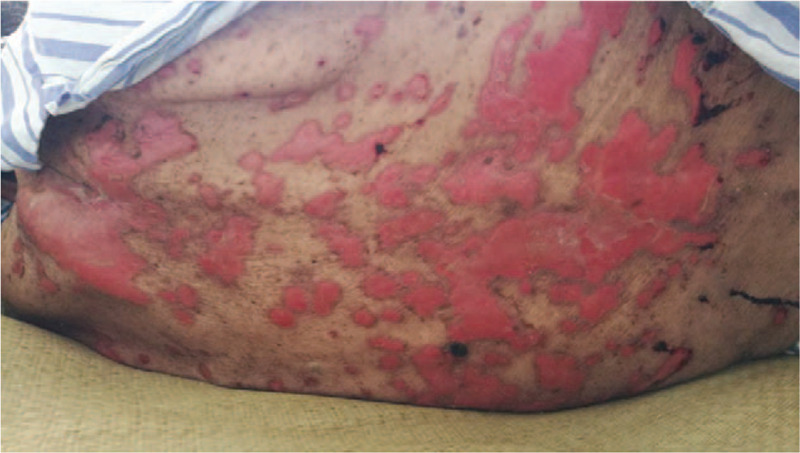
The skin lesions were mitigated and the impaired skin on the back was improved and extensively exfoliated.

Treatment was switched to methylprednisolone 40 mg every 12 hours, intravenous immunoglobulin 10 g once a day for 3 days and ibrutinib 420 mg daily. At the same time, meropenem and fluconazole were used for a possible secondary skin infection. After this regimen, the skin lesions were mitigated and the impaired skin on the back was improved and extensively exfoliated (Fig. 3). Subsequently, methylprednisolone was slowly reduced to 30 mg daily and maintained, with other treatments unchanged. Six weeks later, the patient's symptoms improved remarkably and ELISA indices for Dsg3 and BP180 decreased (Dsg3, 32; BP180,70.44). Unfortunately, the patient developed dyspnea due to the lung impairment from SLL and infection 2 months later. Gradually, respiratory failure appeared and mechanical ventilation was requested. Eventually, the patient could not afford the costly treatment and chose to be discharged from the hospital. Thereafter, the patient lost the opportunity for follow-up treatment.

## 3 Discussion

PNP is a severe life-threatening autoimmune blistering disease associated with underlying lymphoproliferative disorders. Etiopathogenesis of PNP has not been fully described. Skin lesions are thought to originate from an antibody-mediated autoimmune response to tumor antigens that cross-react with epithelial antigens including plakin family proteins, desmogleins, desmocollins and bullous pemphigoid antigens.^[3]^ The cutaneous lesions are polymorphic and the clinical manifestation may resemble pemphigus, pemphigoid, erythema multiforme, graft-versus-host disease, or lichen planus.^[3]^ Hence, the diagnosis of PNP can be extremely easily misdiagnosed. Therefore, when severe, stubborn stomatitis and skin lesions appeared, the possibility of PNP should be considered. The overall prognosis of PNP is poor, and the mortality rate is very high.^[7]^ Patients often die from complications such as respiratory failure, secondary infection, and multiple organ dysfunction.^[3]^

In this case, the earliest symptoms of PNP begin with skin erythema. But in most cases, skin lesions tend to follow oral mucosal lesions. Stomatitis occurs in almost all patients with PNP. The mucosal lesions progressively worsen with disease progression and are extremely resistant to treatment.^[8,9]^ Persistent, extensive, and severe mucosal lesions are important clues to the diagnosis of PNP.^[3]^ Currently, there is no consensus about the diagnostic criteria for PNP; the diagnosis is based on the criteria of Anhalt et al mostly on clinical and histologic observations, immunofluorescence, enzyme-linked immunoassays and immunoprecipitation tests.^[1,3]^ Immunoprecipitation is the most sensitive and specific test for measuring anti-plakin antibodies in PNP.^[3,10]^ A positive immunoprecipitation test is considered the gold standard criteria for the diagnosis of PNP.^[3,11]^ However, it has limited availability. Alternatives for the detection of plakin autoantibodies include immunoblotting and enzyme-linked immunoassays.^[3]^ Thus, in this case, PNP was diagnosed according to the clinical manifestation, histologic observations, immunofluorescence and enzyme-linked immunoassay tests.

Nowadays, the first line of treatment is immunosuppression with high doses of systemic corticosteroids.^[12]^ Other immune suppressants used in the treatment are cyclophosphamide, cyclosporine, and tacrolimus and azathioprine.^[12]^ In addition, early detection and treatment of underlying tumors are very important; biological agents such as high-dose intravenous immunoglobulin are not very effective; Plasma exchange can reduce the autoantibodies produced by the tumor.^[3]^ In this case, methylprednisolone was given to suppress the immune system, intravenous immunoglobulin was administered to regulate immunity and ibrutinib was used for the underlying tumor. At the same time, meropenem and fluconazole were used for a possible secondary skin infection caused by the loss of skin integrity and the use of potent immunosuppressant. Patients with PNP have an increased susceptibility to skin infections related to the loss of skin integrity and the use of potent immunosuppressant. Therefore, early treatment of secondary infections with proper systemic antimicrobial therapy is of significant relevance to prevent sepsis and death.^[3,13]^ After a series of treatments, the skin lesions were prominently improved and ELISA indices for Dsg3 and BP180 decreased. Unfortunately, the patient eventually developed respiratory failure and he chose to be discharged from the hospital. After that, the patient lost the opportunity for follow-up treatment.

In conclusion, when patients with SLL or other neoplastic diseases present with erythema skin lesions, intractable stomatitis or other mucosal lesions, the possibility of PNP should be considered and ancillary testing should be implemented promptly. The most important thing is that the skin lesions are polymorphous, and we should pay great attention to differential diagnosis to prevent misdiagnosis.

## Author contributions

Zhilu Chen made substantial contributions to conception and design. Dafen Wang drafted the manuscript and revised it critically for important intellectual content. Both authors gave final approval of the version to be published.

## Abbreviations

Abbreviations: BP180 = bullous pemphigoid antigen 180, Dsg3 = desmoglein 3, ELISA = enzyme-linked immunoassays, PNP = paraneoplastic pemphigus, SLL = small lymphocytic lymphoma.
